# Effects of cyclone-generated disturbance on a tropical reef foraminifera assemblage

**DOI:** 10.1038/srep24846

**Published:** 2016-04-29

**Authors:** Luke C. Strotz, Briony L. Mamo, Dale Dominey-Howes

**Affiliations:** 1 Department of Geology and Geophysics, Yale University, New Haven, CT, USA; 2 School of Biological Sciences, The University of Hong Kong, Hong Kong SAR, China; 3 Asia – Pacific Natural Hazards Research Group, School of Geosciences, The University of Sydney, Sydney, NSW, 2006, Australia

## Abstract

The sedimentary record, and associated micropalaeontological proxies, is one tool that has been employed to quantify a region’s tropical cyclone history. Doing so has largely relied on the identification of allochthonous deposits (sediments and microfossils), sourced from deeper water and entrained by tropical cyclone waves and currents, in a shallow-water or terrestrial setting. In this study, we examine microfossil assemblages before and after a known tropical cyclone event (Cyclone Hamish) with the aim to better resolve the characteristics of this known signal. Our results identify no allochthonous material associated with Cyclone Hamish. Instead, using a swathe of statistical tools typical of ecological studies but rarely employed in the geosciences, we identify new, previously unidentified, signal types. These signals include a homogenising effect, with the level of differentiation between sample sites greatly reduced immediately following Cyclone Hamish, and discernible shifts in assemblage diversity. In the subsequent years following Hamish, the surface assemblage returns to its pre-cyclone form, but results imply that it is unlikely the community ever reaches steady state.

Tropical reef systems are important refugia for biodiversity, harbouring an array of critical and charismatic taxa, and have both significant cultural and economic value^1^. The high-intensity winds, waves, torrential rains, and flooding associated with tropical cyclone events result in high levels of damage to tropical reef communities^2^,^3^ through direct physical destruction^4^,^5^ and increased sediment input and suspended sediment residence times^6^,^7^,^8^. Because of these concerns, there is growing interest in methods to reliably predict the frequency and intensity of tropical cyclone events^7^. Much of the work in this area has focused on predictive-modelling approaches based upon meteorological data^9^,^10^. A number of studies have demonstrated however, that assessing future hazards begins with and requires an understanding of a region’s hazard history^11^,^12^,^13^,^14^.

One important proxy for quantifying the tropical cyclone event history of a region is the sedimentological record. Previous research has identified past cyclone and hurricane events through investigation of sedimentary dynamics and microfossil distributions^6^,^15^,^16^,^17^. This work has concentrated on the assumption that the presence of a distinct, temporally brief, allochthonous sedimentary unit or microfossil assemblage is conclusive evidence of a palaeo-cyclone or hurricane^12^,^13^,^14^,^15^,^17^,^18^. In practice, this means identifying storm-transported sediments or species from deeper water deposited in a shallow-water or terrestrial setting^19^. Whilst this method has been employed widely and successfully, the dynamics of storm event deposition is still incompletely understood^20^. Work on known hurricane events in North America has identified variation in signal character and quality for allochthonous sedimentary and microfossil material^12^,^13^,^14^,^18^,^19^,^21^,^22^,^23^,^24^,^25^,^26^,^27^,^28^ associated with tempestite deposits, and that only sites in close proximity to high-intensity storms may yield the expected allochthonous signal^20^. Because the possibility has not been extensively explored, it is also conceivable that currently unknown signal types, indicative of high-intensity storm activity and that differ from the expected allochthonous assemblage, may exist. If the latter is the case, the frequency of hurricane and cyclone events is potentially underreported.

The goal of our study is to address some of these uncertainties through comprehensive examination of microfossil samples collected before and after a known tropical cyclone event. We focus on foraminifera from Heron Reef, located at the southern end of the Great Barrier Reef (GBR). In comparison to the large body of work using foraminifera as ecological indicators, the potential of foraminifera in natural hazard investigation is only just beginning to be explored^11^. Previous foraminifera-hazard studies have concentrated on sub-tropical and temperate siliciclastic settings^12^,^13^,^14^,^15^,^17^,^18^. Fewer studies have used foraminifera to quantify regional hazard histories in tropical carbonate settings^29^,^30^.

The Australian Bureau of Meteorology lists four Category 5 tropical cyclones and six severe tropical cyclones of lesser intensity that have impacted the GBR since 2006. Our event is Cyclone Hamish (2009), a Category 5 tropical cyclone at its maximum intensity, but a Category 4 tropical cyclone when it reached Heron Reef (Fig. 1). As we had previously collected foraminifera samples from Heron Reef in 2008, prior to the impact of Cyclone Hamish, this afforded a rare opportunity to characterise a disturbance assemblage generated by a known tropical cyclone and contrast it with a commensurate ‘pre-event’ assemblage.

Our results do not identify any evidence that Cyclone Hamish created an allochthonous foraminifera assemblage consistent with that identified for other similar events. This means that Cyclone Hamish would not be detected in the sedimentary record of Heron Reef using traditional approaches. However, we are able to highlight a range of signals that we can directly attribute to our known tropical cyclone event by employing a suite of techniques typical of ecological studies but rarely employed to address questions in the geosciences (see Methods). The combination of signals we find have not been previously recognised in tropical cyclone studies and compliment the allochthonous assemblage approach. We conclude by considering the implications of our result for future assessments of tropical cyclone histories using the sedimentary record.

## Results

Species occurrences and abundances for all samples are presented in Supplementary Table 1. SEM micrographs of key species are provided in Fig. 2.

A traditional ecological approach to assessing disturbance would examine changes in both community composition and diversity, as the effects of the tropical cyclone on the biota may be uniform or may only impact specific species. We take this approach here. It would also use a comparison between an undisturbed ‘control’ and a disturbed ‘treatment’, both sampled at a coeval interval. Due to the size of Cyclone Hamish, a ‘control’ site would need to be hundreds of kilometres from the ‘treatment’ locality. This would create its own difficulties, as dissimilarity in species composition and abundance would reflect regional differences or local conditions^31^ rather than disturbance. Instead, we therefore employ the assumption that initial sampling in 2008 represents a pre-cyclone control and that all subsequent sampling (2009–2011) represents cyclone-disturbed treatments and recovery assemblages. A similar assumption has been used in palaeo-hazard studies, where a potential disturbance assemblage down-core is compared to surrounding undisturbed ‘background’ assemblages^12^,^17^. We discuss the implications of relaxing this assumption in the Discussion.

Using the total dataset, there is no significant difference in community composition between the four sampling periods (PERMANOVA: *F* = 0.653, *P* = 0.662). PCoA yields a similar result, with overlap for the four sampling intervals (Fig. 3), a result we interpret as representative of a single grouping. The reef flat foraminifera assemblage is dominated by *Calcarina hispida* (70% of the assemblage), *Baculogypsina sphaerulata* (~12%) and *Marginopora vertebralis* (2%). This order of dominance is repeated for all sample intervals and at all reef flat sample sites, with the exceptions of H17 and H18 (Supplementary Table 1). At H17 and H18, *B. sphaerulata* is the most prevalent taxon, followed by *C. hispida*. These dominant taxa belong to the informal ‘Larger Benthic Foraminifera’ group; heavily calcified taxa typical of tropical reefs throughout the Pacific^32^,^33^. There are a large number of rare taxa (74 taxa <1% relative abundance in any sample), producing the ‘hollow curve’ characteristic of many species rank abundance plots^34^ (Fig. 4). Rank abundance curves are similar for all sampling intervals (ANOVA: F = 0.000004, *P* = 1; Tukey HSD: all pair-wise *P* > 0.05). These results provide no evidence for a disturbance assemblage associated with Cyclone Hamish.

Nor do we find any evidence of allochthonous material transported from deeper water onto the reef flat. The foraminifera biota in the Wistari Channel sample is distinctly different to the reef flat assemblage. The most prevelant taxa are *Amphistegina lessonii* (17%), *Heterostegina depressa* (11%) and *Assalina ammonoides* (10%). *Calcarina hispida* makes up only 5% of the assemblage and *B. sphareulata* is absent. Species richness is higher in the channel than for any reef flat sample (55 species – Supplementary Table 1). Examining the four reef flat sampling intervals, there is no change in *A. lessonii* or *H. depressa* abundance from 2008 to 2009 (*A. lessonii*_Mvabund_ *P* = 0.633; *H. depressa*_Mvabund_ *P* = 0.398). *Assalina ammonoides*, along with six other taxa exclusive to Wistari Channel, are not in any of the reef flat samples from any sampling interval.

A more detailed examination of our PCoA however, focusing on PCoA distance values for the first two eigenvectors (PCO1 and PCO2), provides potential indicators of disturbance. Box-plots of PCO1 distance values reveal a narrowing of the interquartile range (IQR) for the 2009 sampling interval, when compared with 2008, and a gradual return to the pre-cyclone IQR over the following two sampling intervals (Fig. 5). They also highlight the presence of potential outliers in the dataset. Using Grubbs test, we confirm two samples, H17 and H18, as outliers for all four sampling intervals. For PCO2, we identify a reduction in the range of distance values for the 2009 sampling period when compared with 2008 (PCO2range_2008_ = 0.511, PCO2range_2009_ = 0.245; 52% reduction). In 2010, the range increases (PCO2range_2010_ = 0.414) and by 2011 the range is slightly higher than the 2008 value (PCO2range_2011_ = 0.529). There is no comparable reduction in distance value range on PCO1.

When we repeat PERMANOVA with outliers removed for all sampling intervals, we obtain a significant difference in community composition across the four sampling periods (*F* = 2.305, *P* = 0.008). We interpret this as evidence of disturbance. Crucially, in a pair-wise comparison between 2008 and 2009 we obtain a significant result (*P*_08–09_ = 0.04). There is no change from 2009 to 2010 (*P*_09–10_ = 0.137) and 2008 and 2011 are comparable (*P*_09–10_ = 0.3352) but there is a difference between 2010 and 2011 (*P*_10–11_ = 0.031). Excluding taxa with <0.1% total relative abundance (reducing our dataset to 34 taxa) reveals our overall result is sensitive to the presence/absence of rare taxa (*P* = 0.02) but is still significant.

Mvabund analysis of deviance identifies changes at the community level across the sampling intervals (*P* = 0.001, outliers removed). Twenty-one taxa have significant changes in their abundance across the four sampling intervals. We only consider two of these results *potentially* valid: *Cymbaloporetta bradyi* (*P* = 0.041) and *Rudigaudryina minor* (*P* = 0.016). Both these taxa exceed 50 individuals in at least two sampling intervals. For all other taxa with significant results, this threshold is not reached and we do not consider the sample size statistically valid. The observed changes in the abundance of these two taxa parallel the changes in PCoA distance values, PERMANOVA and diversity. The relative abundance of *C. bradyi* and *R. minor* are both halved between the 2008 and 2009 sampling intervals (Supplementary Table 1). By 2011, both return to 2008 levels. The two taxa do not share a common morphology, nor is their distribution exceptional compared with other taxa or each other.

Diversity analyses provide further evidence of disturbance. Supplementary Table 2 contains diversity results for each sample site. At the majority of sites there is a decrease in values for *q* = 0, *q* = 1 and *q* = 2 post-cyclone and a return to pre-cyclone values over the subsequent two sampling intervals. At the assemblage level, there is an overlap of confidence intervals for *q* = 0 for all sampling intervals, although overlap between 2011 and other sampling intervals is only obtained through extrapolation of all sampling intervals to double the reference sample size (Fig. 6). Shannon and Simpson diversity results (*q* = 1 and *q* = 2) parallel each other. Both are asymptotic in all sampling intervals, with no increase in values even when extrapolated to double the reference sample size. Both values decrease from 2008 to 2009, with no overlap of confidence intervals; both return in 2010 to values comparable to 2008, with confidence interval overlap and both reach values in 2011 that exceed all other sampling intervals with no confidence interval overlap. For a fixed sample size, where confidence intervals for Hill number rarefaction/extrapolation do not overlap, significant difference (at a level of 5%) is guaranteed^35^. These results highlight a decrease in both evenness and overall diversity in the 2009 samples, with values returning to, and then exceeding, 2008 values over the subsequent two years. The overlap in species richness indicates these changes largely represent fluctuations in abundance. The rapid levelling off of Shannon and Simpson diversity in all sampling intervals (Fig. 6) is additional evidence (along with the steep rank abundance plot – Fig. 4) of the strong dominance of common species.

Values for Pielou’s Evenness Index (Fig. 7) parallel those of PCoA distance values (Fig. 5) and Shannon diversity (Fig. 6). A decrease in the mean and a narrowing of the IQR is identified immediately after Cyclone Hamish. In subsequent sampling intervals mean evenness increases above initial values (similar to the pattern observed for *q* = 1) and the IQR returns to values comparable to 2008.

There is no significant taphonomic signal. For pair-wise comparisons of the four sampling intervals, Tukey HSD yields no significant values for breakage or surface alteration (all pair-wise *P* > 0.05). Even when considering Larger Benthic Foraminifera or the remainder of the assemblage (‘smaller foraminifera’) separately, there is no significant difference between sampling intervals.

We detect no difference in grain size across the four sampling intervals (PERMANOVA: *F* = 0.857, *P* = 0.504; all pair-wise *P* > 0.05). Any differences in the foraminifera assemblage detected through our analyses are thus not due to changes in grain size.

## Discussion

Changes in the foraminifera assemblage are most evident immediately following Cyclone Hamish. The Australian Bureau of Meteorology lists Cyclone Rewa in 1994 as the last cyclone to pass over Heron Reef prior to Cyclone Hamish. Since 1999, no cyclone has passed with 450 kilometres of Heron Reef. For both the period of sampling (2008–2011) and the proceeding two years (2006–2007), the number of cyclones and number of severe cyclones (a minimum central pressure less than 970 hPa) in the Australian region was below the regional average (Supplementary Figure 1). Four severe tropical cyclones (Cyclones Laurence, Ului, Zelia and Yasi) and four cyclones of lesser intensity occurred in the north-eastern Australian region during the period of post-Hamish sampling (May 2009 – April 2011). The long period of cyclone inactivity in the direct vicinity of Heron Reef, the consistent (and diminished) background cyclone activity, the clear temporal association between the observed changes in the foraminifera assemblage and Cyclone Hamish and the lack of similar effects on the foraminifera assemblage associated with severe but more distant cyclone events precludes the possibility that our results are the product of a regional cyclone event or events.

The Australian Institute of Marine Science weather station on Heron Reef records minimal variation in sea surface temperatures for the four sampling intervals (between 25.3 and 25.7°) and what variation does exist does not align with the changes we observe in the foraminifera biota. According to the Australian Bureau of Meterology, the only climate variable that matches our results for the relevant temporal period is wind speed, a direct reflection of the high intensity winds associated with Cyclone Hamish. This means we can rule out the possibility our signals are the result of short term fluctuations in climatic drivers (except changes in those factors directly linked to Cyclone Hamish).

The most credible explanation for the observed changes in the Heron Reef foraminifera assemblage across the four sampling intervals is therefore a tropical cyclone generated disturbance (directly attributed to Cyclone Hamish) and an associated recovery. In the immediate aftermath of Cyclone Hamish, we identify an increased similarity between sample sites compared to what existed pre-cyclone, and an associated reduction in diversity. Over the following two sampling intervals, the community returns to a pre-cyclone like assemblage. Due to community stochasticity^36^, we expect small fluctuations in diversity between sampling intervals, regardless of any disturbance event. However, we account for these small fluctuations by undertaking analyses at the assemblage level. The decreased disparity and subsequent recovery identified by our analyses is consistent and significant across multiple analytical results, emphasising the robustness of our findings. The observed homogenisation of the assemblage is attributed to the extreme wave and current forces associated with a tropical cyclone, known to entrain and transport reef sediments^5^. In shallow tropical reef flat environments, foraminifera assemblages are coarsely zonated, based upon distance from the reef rim and inter-reef circulation patterns^31^,^37^. Entrainment and transport of sediments by tropical cyclones must invariably result in sediment mixing, breaking down the existing pattern of zonation, and leading to homogenisation of the foraminifera assemblage. Some destruction of tests must also take place during this process to explain the diversity decrease that occurs. Destruction must be confined to rare taxa, as there is no significant change in the abundance of dominant taxa (especially the robust ‘Larger Benthic Foraminifera’), and it must result in complete destruction of the test, as there is no significant change in values for test breakage or surface alteration.

We identify these impacts of Cyclone Hamish without any evidence of deeper-water allochthonous taxa present in the post-cyclone sampling interval. If we had only focused on taxon level signals, it would have been insufficient to detect significant variation between the pre- and post-cyclone sampling intervals. We record no change in the most abundant taxa, and only tentatively propose a disturbance-based change for two taxa (*C. bradyi* and *R. minor*). For all other taxa, their extreme rarity makes it equally plausible that changes in abundance are due to sampling bias associated with the number of individuals sampled. Even the methods we use to evaluate variation at the assemblage level (e.g. PERMANOVA; Mvabund) yield no result if applied to the total assemblage without consideration. Only using multiple techniques, accounting for outliers, and most crucially, focusing on the assemblage as a whole, is a discernible signal identified.

Importantly, the observed disturbance signals are distinct from a typical foraminifera response to environmental change, which largely consists of either assemblage and/or abundant species turnover^38^. We therefore propose we have identified new signals of tropical cyclone disturbance distinct from the signals identified in previous work. The methods we have used are complimentary to the methods used to identify storm-transported material, and both signal types can be established using the same dataset. If our signals are general ones, they should be discernible in the sedimentary record, as the analysis methods used herein can easily be applied to comparison of down-core microfossil assemblages. As foraminifera are prolific on tropical reefs globally^32^,^33^, our methods can be applied to any tropical reef system impacted by tropical cyclones. The observed homogenisation/diversity change response is perhaps only possible because of the unique nature of microfossils, representing both an organism and a sedimentary particle. We are not aware of any tropical cyclone study where homogenisation combined with a diversity decrease has been observed. A previous study of displaced foraminifera associated with Hurricane Irene found homogenisation, but not the same changes in diversity^20^. Previous studies examining the effects of cyclone generated- disturbance on other organisms, for example corals^39^,^40^,^41^ or macroalgae^42^,^43^, identify fluctuations in species richness, the proliferation of disaster taxa (either survivors or opportunists) and varying rates of recovery, but no homogenisation.

Because the presence of an allochthonous assemblage is commonly used to identify past storm activity in the sedimentary record, our results have implications for paleotempestology. Cyclone Hamish, a Category 4 event, would be completely undetected on Heron Reef if looking only for an allochthonous assemblage. That an event of this magnitude does not produce the expected signal further highlights that our understanding of storm-related deposition is not definitive^20^,^44^. There are however, a number of caveats to our results that need to be addressed before our findings can be directly applied to quantifying regional hazard histories.

First, our results are based upon a single Category 4 event. Replication of our results for other cyclone events is needed to confirm the generality of our signal. It is entirely possible that our signal is specific to tropical carbonate settings, or even to this one tropical cyclone event. Because our study was opportunistic, predicated on a tropical cyclone impacting a previously collected site, repeated replication of our exact methods may be problematic. Perhaps an easier test is to examine already identified cyclone deposits from the sedimentary record (fingerprinted using allochthonous material). If our signal is a general one, then it should be observed in those deposits as well.

**Figure 1:**
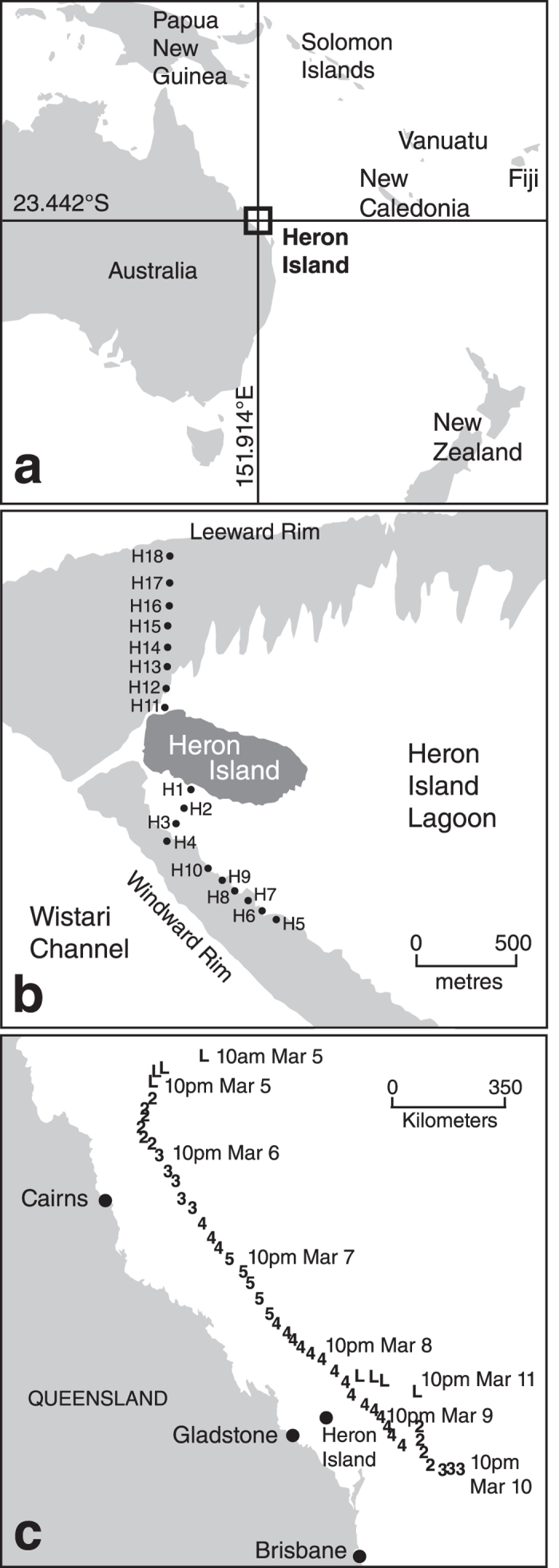
Map of Heron Island and path of Cyclone Hamish. (**a**) Location of Heron Island. (**b**) Heron Island and Heron Reef flat, showing location of sample sites. (**c**) Path of Cyclone Hamish from formation to dissipation. Times and dates indicate location of Hamish at that instance. Numbers indicate both path of Hamish’s ‘eye’ and its intensity at that point in time (intensity values based upon Australian tropical cyclone intensity scale). (**a**,**c**) Generated in R.3.1.1^67^ using the ‘maps’ package (version 3.1.0)^68^. (**b**) Created with Adobe Illustrator CS3. Data for (**c**) comes from Joint Typhoon Warning Center operational warnings and advisories.

**Figure 2:**
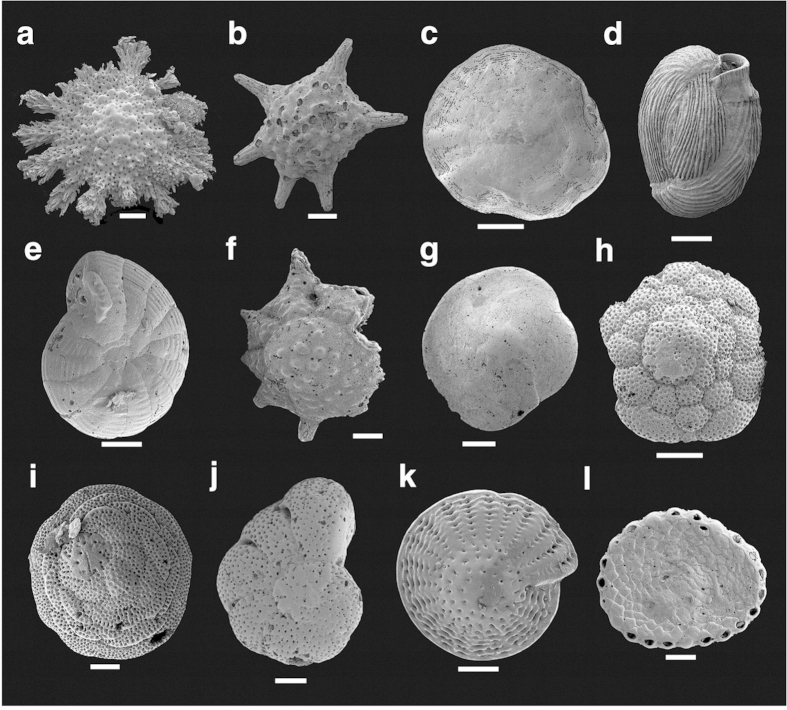
Dominant foraminifera taxa from Heron Reef flat across the four sampling intervals. Plate shows 12 most abundant species in rank abundance order. All illustrated specimens from 2008 interval. Full species name, specimen number, specimen view and length of scale bar provided for each specimen. (**a**) ***Calcarina hispida*** Brady, MU 63014, aboral view, scale bar = 200 μm. (**b**) ***Baculogypsina sphaerulata*** (Parker and Jones), MU 63009, lateral view, scale bar = 200 μm. (**c**) ***Marginopora vertebralis*** Quoy and Gaimard, MU 62931, lateral view, scale bar = 2 mm. (**d**) ***Quinqueloculina neostriatula*** Thalmann, MU 62867, lateral view, scale bar = 200 μm. (**e**) ***Peneroplis pertusus*** (Forskål), MU 62920, lateral view, scale bar = 100 μm. (**f**) ***Neorotalia calcar*** (d’Orbigny), MU 63007, aboral view, scale bar = 100 μm. (**g**) ***Amphistegina lobifera*** Larsen, MU 63003, aboral view, scale bar = 200 μm. (**h**) ***Cymbaloporetta bradyi*** (Cushman), MU 62982, aboral view, scale bar = 100 μm. (**i**) ***Millettiana milletti*** (Heron-Allen and Earland), MU 62985, aboral view, scale bar = 100 μm. (**j**) ***Epistomaroides punctatus*** (Said), MU 62995, lateral view, scale bar = 100 μm. (**k**) ***Elphidium craticulatum*** (Fichtel and Moll), MU 63018, lateral view, scale bar = 200 μm. (**l**) ***Sorites orbiculus*** Forskål, 1775, MU 62934, lateral view, scale bar = 100 μm. All SEM images taken by the authors.

**Figure 3:**
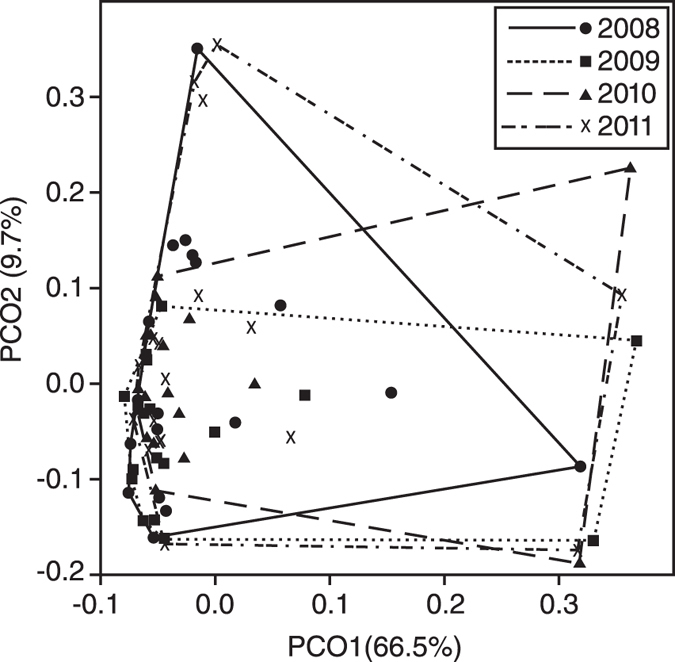
Principal Coordinates Analysis biplot for foraminiferal assemblages from Heron Reef flat. Symbols represent sample sites. Convex hulls outline each of the four sampling intervals (2008–2011). Numbers in brackets indicate % variance attributed to that axis.

**Figure 4:**
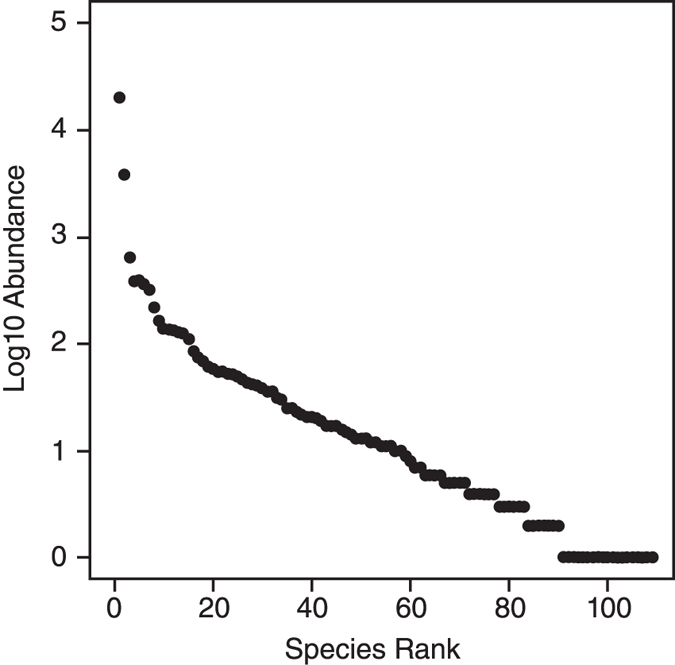
Rank abundance plot (Whittaker plot) for total foraminiferal assemblage (all four sampling intervals combined) from Heron reef-flat. The observed pattern is identical for each sampling interval when assessed individually.

**Figure 5:**
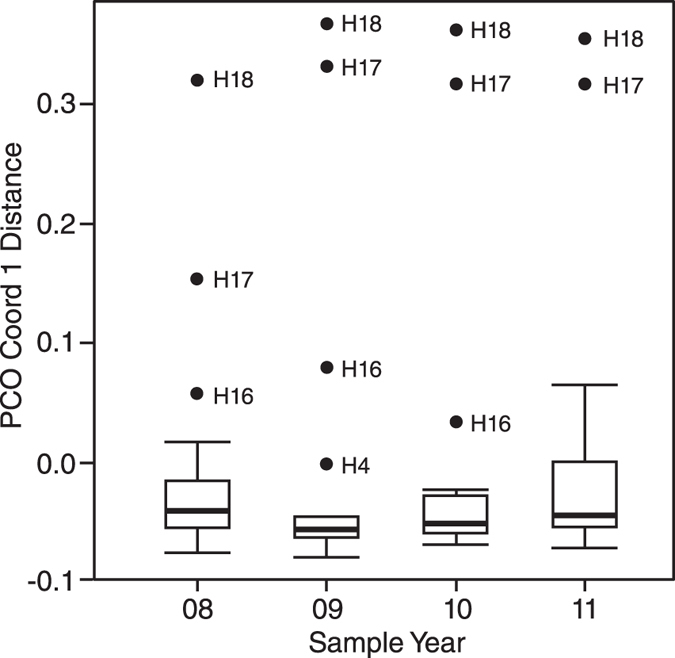
Box plot of PCoA Coordinate 1 distance scores for each of the four sampling intervals. Band inside box = median. Whiskers = +/−1.5*Interquartile range (IQR) and points represent sample sites that fall outside whiskers. Note the reduced IQR directly after Cyclone Hamish (2009), and the broadening of the IQR in subsequent years.

**Figure 6:**
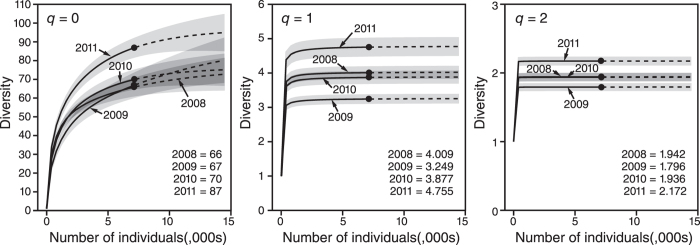
Sample-size-based rarefaction (solid line) and extrapolation (dashed lines up to double the reference sample size) of foraminiferal diversity for Hill numbers. *q* = 0 (left panel), *q* = 1 (middle panel), and *q* = 2 (right panel). Reference sample for each sampling interval is indicated by a solid dot and the calculated Hill numbers are listed in the bottom right corner. Across the four sampling intervals, total foraminifera species richness (*q* = 0) equals 109 taxa. The result of extrapolation of total species richness for double the reference sample size is 122 taxa and has not yet reached the asymptote.

**Figure 7:**
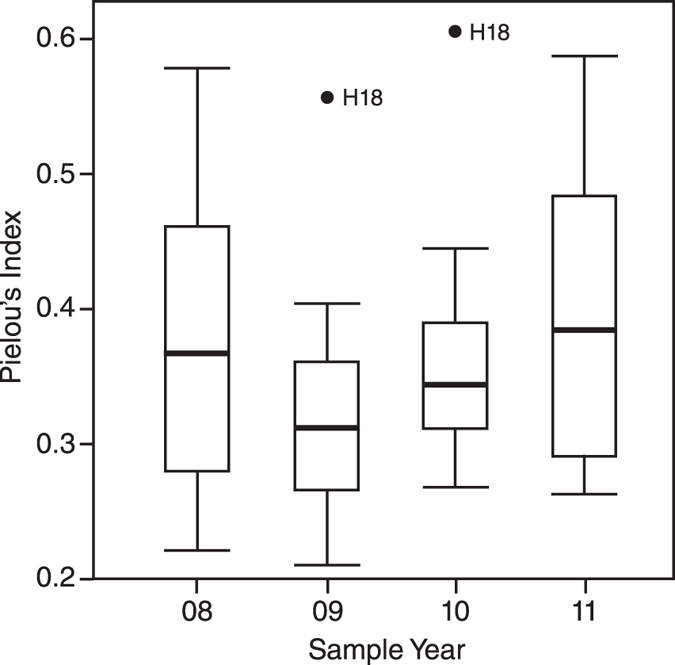
Boxplot of Pielou’s Evenness Index for the four sampling intervals. Band inside box = mean. Whiskers = +/−1.5*Interquartile range (IQR) and points represent sample sites that fall outside whiskers.

Second, the presence of outliers in the dataset indicates that, at some localities, the assemblage may not yield a discernible signal when one may exist elsewhere. In our case, our outliers are located on the leeward reef rim, the direction of approach for Cyclone Hamish (Fig. 1), and would have borne the full brunt of the cyclone. We identify neither an allochthonous assemblage nor any of our new signals at these sites. We cannot explain why we record no evidence of impact at these sites, but the lack of a discernible signal associated with a known storm event is not exclusive to our study^20^. These results highlight that site selection may be key to identifying palaeo-cyclone activity in the sedimentary record.

Third, we use the assumption that the pre-cyclone sampling interval represents an ‘undisturbed’ assemblage. Because values for diversity in 2011 exceed 2008 values, this assumption could potentially be relaxed, and 2008 identified as ‘less disturbed’. The lack of a completely stable baseline does not invalidate our result, as our focus is on identifying changes between pre- and post-cyclone sampling intervals and does not require that the pre-cyclone sampling interval represents a climax community, but raises the possibility that the community was not at steady state when initially sampled. The lack of a stable climax assemblage is not necessarily surprising. Previous work has identified that coral reef communities are subject to phase-shifts, rather than alternative stable states in association with disturbance^45^. Whilst tropical cyclone activity in the direct area around Heron Reef was limited in the decade prior to sampling, even this length of time may not be enough for the community to rebound from the impact of a tropical cyclone and reach a climax assemblage. Other organisms show varying rates of recovery in coral reef settings, with up to 70 years required for some coral populations to return to a pre-event type community^46^. For all intents and purposes, tropical systems are permanently in a state of intermediate recovery. This is important to our result as, if tropical reef foraminifera communities are rarely at steady state, potentially only severe palaeo-tropical cyclone or palaeo-tropical cyclones that occurred after a long period of no tropical cyclone activity may be detectible in the sedimentary record using our methods, as other events will resemble the background community.

There is currently no way to be certain that the signal we have identified is preserved into the longer-term sedimentary record. Homogenisation of the assemblage as a direct result of hurricane based disturbance has been previously observed in core material, suggesting preservation of the type of signals we observe is possible, but those results were from a siliciclastic setting^20^. There are known issues with bioturbation and sediment disturbance in carbonate reef settings that affect the likelihood that our signal is preserved at depth^47^. However, reef sediments are still considered applicable to sub-recent palaeoecological studies^48^, and there are demonstrated cases where palaeo-ecological signals are preserved into the longer-term sedimentary record in reef environments^49^,^50^,^51^, including studies using foraminifera^52^. Only extensive coring can resolve whether the type of microfossil signals we find associated with Cyclone Hamish can be preserved into the sedimentary record in a carbonate setting. Because of this uncertainty, the direct applicability of our result to palaeo-tropical cyclone studies remains currently unknown. What our results do clearly reveal is that a major cyclone event may not always generate the expected allochthonous deposit or assemblage and that there are other potential signals of palaeo-tropical cyclone activity. We encourage future investigations to investigate the potential generality of our signal, as these can only enhance estimates of tropical cyclone frequency-recurrence and generate better resolved regional histories of tropical cyclone activity.

## Methods

### Study Area

Heron Reef (Fig. 1) is a 9.5 km by 4.5 km wide lagoonal reef platform with a vegetated cay on the western leeward side, a well-developed windward rim to the south and a less well-developed northern leeward rim^50^. Heron Reef displays a well-developed, concentric zonation from its reef-slope to back-reef and lagoonal areas, broadly divided into shallow (tidally influenced) and deep (sub-tidal) areas^53^. The sediments deposited across Heron Reef are composed almost exclusively of calcareous skeletal material. Major components include fragments of coral, molluscs and calcareous algae^50^. Foraminifera are only a minor component of the deposited sedimentary material (1–12%)^37^.

Cyclone Hamish formed on the 4^th^ of March 2009 and by the 7^th^ of March had reached Category 5 intensity (Fig. 1c). On the 9th of March at approximately 10 pm (AEDT), after travelling in a south-easterly direction parallel to the Queensland coastline, Cyclone Hamish swept over Heron Reef at Category 4 intensity.

### Data collection

This study is based upon repeated collection of surface material from eighteen reef-flat spot localities on the southern and north-western parts of Heron Reef. Samples were collected in April 2008, in March 2009 immediately following Cyclone Hamish, in March 2010 and in April 2011. We also include one deeper water sample, collected only in 2008, from 35 metres depth in Wistari Channel (Fig. 1b). We include this sample as previous studies have used the presence of allochthonous deeper-water taxa as a marker to fingerprint storms^13^,^16^,^17^,^18^.

For repeat sampling post-cyclone, GPS and field observations were used. Reef flat samples (Mean High Water <2 metres) were collected by hand at low tide using a film canister-sized container to sample the top 0.5 cm of sediment (~30–50 g). The Wistari Channel sample was collected using a ponar grab sampler. Since each sample set was collected at the same time of year, we avoid the possibility of seasonal effects on assemblage composition^54^. Samples were washed in freshwater over a 63 μm sieve and oven dried at 50 °C. Dried samples were split with a micro-splitter. Half of the sample was used for grain size analysis and the remaining material examined for foraminifera using standard picking techniques^12^,^13^,^15^. A minimum of 400 foraminifera specimens, following the recommendation of Patterson and Fishbein^55^, were picked and identified from each sample to produce a multivariate data set containing 29,200 individuals. The methods used to produce the data set are comparable to those used in foraminifera palaeo-storm studies. Specimens were taphonomically graded using a method similar to Berkeley *et al.*^56^, but with each specimen assigned two values, one for breakage and one for surface alteration. Grading criteria are outlined in Table 1. Grain size data was generated by passing sediment through a sieve stack (2 mm; 1 mm; 0.5 mm; 125 μm; 63 μm) using a mechanical shaker for 30 seconds. The weight of each size fraction was recorded and then converted to % abundance of total weight (Supplementary Table 3).

### Analysis

Unless specifically noted, all analysis apply to the foraminifera dataset only. Foraminifera raw counts were converted to relative abundances for all analyses. Where the analysis technique included intrinsic data transformation, data was not transformed prior to analysis. For rank abundance curves, abundance data was log10 transformed. For all other analysis, all data was found to be normally distributed using a Shapiro-Wilk test and a Q-Q plot, so untransformed relative abundance data was retained for all analyses. Regardless, we repeated all analyses using natural log and square root transformed data and found comparable results to those generated with untransformed data. Where a similarity measure is required, Bray-Curtis dissimilarity is used^57^. Bray-Curtis has been successfully applied previously to discriminate foraminifera assemblages^32^,^58^,^59^. The Wistari Channel sample is not included in multivariate analysis and is only directly compared to reef flat samples in terms of taxonomic composition and relative abundance.

Principal Coordinates Analysis (PCoA)^59^ is used to visualise dissimilarity between sampling intervals. Using PCoA Coordinate 1 distance scores (PCO1 is the dominant eigenvalue and represents 66.5% of variance), box and whiskers plots were produced based upon the Tukey method^60^, to investigate variation in dissimilarity at the assemblage level across the four intervals. Box plots suggested potential outliers. A Shapiro-Wilk test^61^ was used to confirm that the PCoA coordinate 1 distance dataset is not non-normally distributed and results of a Q-Q plot^62^ suggest these data are normally distributed. Grubbs test^63^ was used to identify outliers. The identified outliers were retained for our initial PERMANOVA analysis but were excluded from all other analyses.

Using one way Permutational Multivariate Analysis Of Variance (PERMANOVA)^64^, with 9,999 permutations and sampling interval as a fixed factor, we assess for significant differences in community composition across the four sampling intervals. PERMANOVA was performed on the total dataset, a dataset where outliers were excluded and a dataset where both outliers and taxa with relative abundance <0.1% were excluded. PERMANOVA was also used to assess changes in grainsize.

The Mvabund approach^65^, based upon a Generalised Linear Model framework (GLM), has been successfully used by its authors to test for significant differences between disturbed and undisturbed experimental treatments and we apply this technique in the same way here. We use this method to test differences in community composition. We also test for taxon-specific differences across sampling intervals by fitting a separate GLM to each species and performing an analysis of deviance for the fitted GLM. We use sampling interval as our explanatory variable at both the community and taxon level.

Changes in diversity across the four sampling periods were identified using diversity accumulation curves with associated rarefaction/extrapolation, calculated using the methods based upon Hill Numbers^35^. Herein we utilise the first three Hill Numbers: species richness (*q* = 0); the exponential of Shannon’s Entropy Index (*q* = 1) and the inverse of Simpson’s Concentration Index (*q* = 2). Extrapolation was undertaken up to double the reference sample size^35^. Pielou’s Evenness Index^66^ was used as a secondary evenness measure to *q* = 1.

Species rank abundance curves were used to look for changes in dominant taxa. Rank abundance and taphonomy results were tested for significant differences across the four sampling intervals using one-way ANOVA and pair-wise comparisons were performed using Tukey’s Honestly Significant Difference (HSD) test. For taphonomy analysis, grading results were first assigned to one of two functional groups, ‘Larger Benthic Foraminifera’ or smaller foraminifera prior to analysis.

All analysis was carried out using R 3.1.1^67^.

## Additional Information

**How to cite this article**: Strotz, L. C. *et al.* Effects of cyclone-generated disturbance on a tropical reef foraminifera assemblage. *Sci. Rep.* **6**, 24846; doi: 10.1038/srep24846 (2016).

## Supplementary Material

Supplementary Information

Supplementary Table 1

Supplementary Table 2

Supplementary Table 3

## Figures and Tables

**Table 1:** Grading scheme used to assess the potential taphonomic effects of Cyclone Hamish.

Breakage Grade	Description	Proportion of test preserved
0	Test is fully intact.	100%
1	Test has limited damage to chamber walls or loss of fragile structures (i.e. broken spines).	>90%
2	More extensive test damage, some chamber loss, but broadly intact.	>70%
3	Test severely damaged, barely recognisable.	>50%
**Surface Alteration Grade**		
0	Test is fully intact.	100%
1	Limited alteration of test surface (i.e. small amount of surface ornament lost).	>90%
2	More extensive alteration to test surface, ornament difficult to distinguish but still present.	>50%
3	Test surface severely altered, ornament unrecognisable.	>25%

Scheme is modelled on method used by Berkeley *et al.*^56^. Each specimen was assigned a grade for both breakage and surface alteration.
