# Supplementary material for: Effects of cyclone-generated disturbance on a tropical reef foraminifera assemblage
Source: Sci Rep. 2016 Apr 29;6:24846. doi: 10.1038/srep24846 (PMC4850380; doi:10.1038/srep24846)

**­Title:** Effects of cyclone-generated disturbance on a tropical reef foraminifera assemblage

**Authors:** Luke C. Strotz, Briony L. Mamo and Dale Dominey-Howes

**Supplementary Table 1** *(In separate excel file)*

**Absolute abundances of foraminifera from Heron Reef flat for the four sampling intervals.**

**Supplementary Table 2** *(In separate excel file)*

**Diversity values for each sample site for four sampling intervals.** Values are for species richness (*q* = 0); the exponential of Shannon’s Entropy Index (*q* = 1) and the inverse of Simpson’s Concentration Index (*q* = 2).

**Supplementary Table 3** *(In separate excel file)*

**Grainsize results for four sampling intervals.** Values represent relative abundance (%) of each size fraction for each sample.

**Supplementary Figure 1**

Tropical cyclone activity in the Australian region 1969 – 2011. Red represents severe events (minimum central pressure less than 970 hPa) and blue represents non-severe events. Data comes from Australian Bureau of Meteorology.


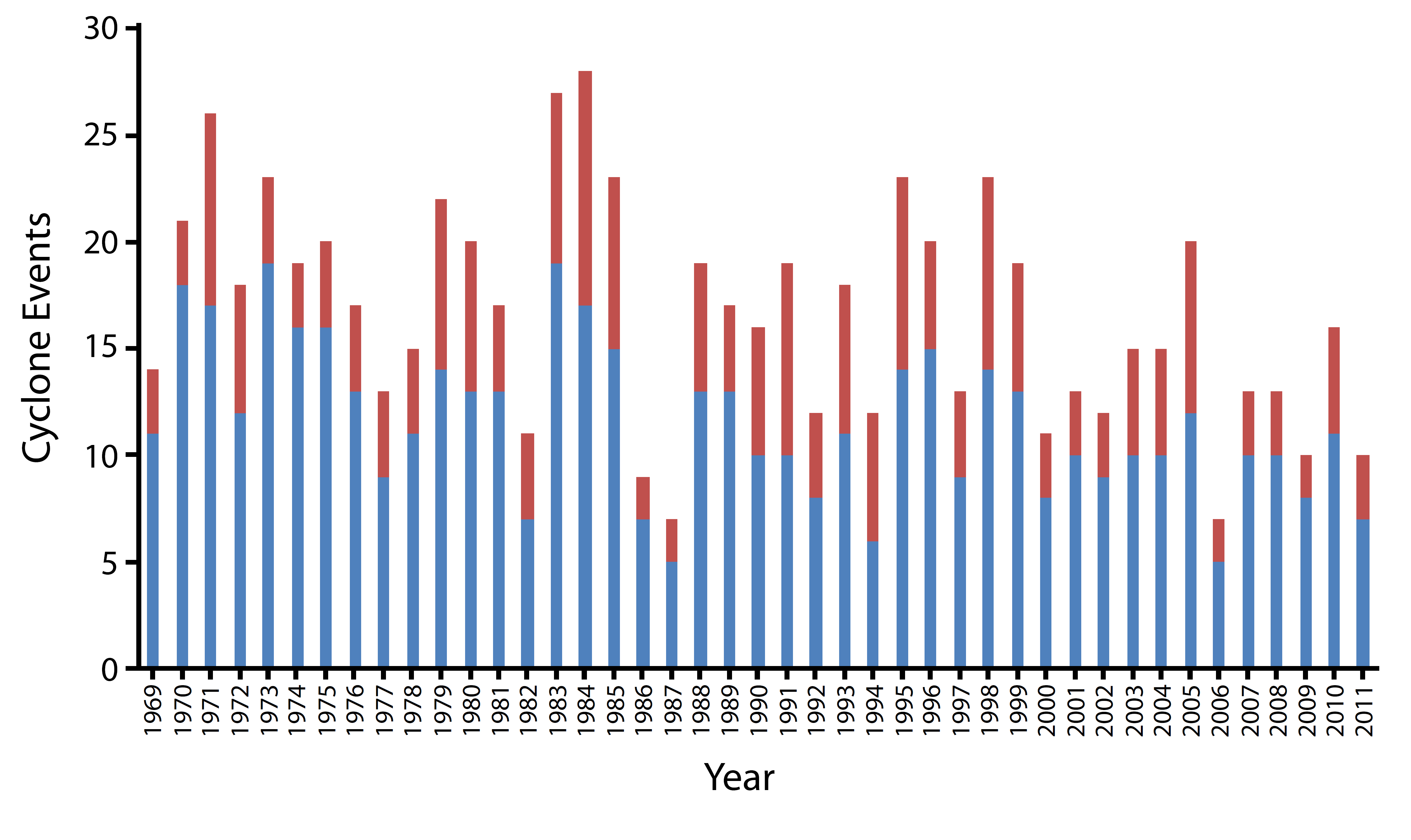

Supplement: Supplementary Information [file srep24846-s1.doc]
